# Special Issue “Cardiovascular Genetics”

**DOI:** 10.3390/genes12040479

**Published:** 2021-03-26

**Authors:** Andreas Brodehl, Hendrik Milting, Brenda Gerull

**Affiliations:** 1 Erich and Hanna Klessmann Institute, Heart and Diabetes Center NRW, University Hospital of the Ruhr-University Bochum, Georgstrasse 11, 32545 Bad Oeynhausen, Germany; hmilting@hdz-nrw.de; 2 Comprehensive Heart Failure Center (CHFC), Department of Internal Medicine I, University Hospital Würzburg, 97080 Würzburg, Germany; Gerull_B@ukw.de

Since the beginnings of cardiovascular genetics, it became evident in thousands of clinical cases that many cardiomyopathies, channelopathies, aortopathies as well as complex multifactorial diseases such as coronary artery disease, atherosclerosis or atrial fibrillation (AF) have a genetic etiology.

In this Special Issue, Ho et al. summarized the genetic relationship between genetic risk factors for cardiovascular diseases and dementia [1]. Vesa et al. showed that single nucleotide polymorphisms (SNPs) in *CYP4F2* (leukotriene-B(4) omega-hydroxylase 1) and *VKORC1* (vitamin K epoxide reductase complex 1) might be risk factors for plaque formation in atherosclerosis [2]. As non-alcoholic fatty liver disease (NAFLD) shares molecular metabolic pathways with atherosclerosis-related cardiovascular diseases (CVDs), Castaldo et al. investigated if NAFLD-associated SNPs might also be related with sub-clinical atherosclerosis [3]. However, the authors excluded an association of specific SNPs involved in NAFLD with atherosclerosis [2]. Peripheral arterial disease (PAD) is frequently caused by the atherosclerosis of leg arteries. In addition to environmental factors such as, e.g., smoking, it is estimated that about 20% of cases with PAD might be influenced by heritable factors [4]. In this context, Renner et al. showed that a specific SNP in the promoter region of the *EPO* gene is associated with an early onset of PAD and could in consequence serve as a potential biomarker [5]. Familial hypercholesterolemia (FH) can be caused by mutations in *LDLR* (low density lipoprotein receptor), *APOB* (apolipoprotein B) and *PCSK9* (proprotein convertase subtilisin/kexin type 9). Meshkov et al. genotyped a large cohort of Russian patients with FH and identified over 200 variants in these genes [6]. The authors reported that over 35% of them are novel and specific for the Russian population [6]. Aortic aneurysms can lead to aortic dissections and contribute to sudden death. Creamer et al. summarized in this Special Issue the genetic and molecular knowledge about hereditary aortopathies [7]. Interestingly, the transcriptomic analysis of human ascending aortic tissue performed by Zhou et al. revealed detailed molecular insights into pathways involved in aortic dissection [8]. In addition to inflammation, the cell death and degeneration of smooth muscle cells, the authors focused on autophagy pathways [6]. The work of Greene et al. defined the transcriptomes of normal aortic valves and those with aortic stenosis (AS) and aortic insufficiency (AI) [9]. Of note, valves with AS and AI have a unique specific gene expression pattern [7].

In clinical practice, cardiomyopathies are classified according to their structural and functional features into dilated (DCM), hypertrophic (HCM), arrhythmogenic (ACM), restrictive (RCM) and left-ventricular non-compaction (LVNC) cardiomyopathies. All cardiomyopathies can be caused by genetic and non-genetic factors including, e.g., myocarditis. In 1990, the first HCM-associated mutation was identified in *MYH7*, encoding myosin heavy chain β [10]. In recent decades, several other HCM-associated genes have been described. The majority of these genes encode sarcomere or sarcomere-associated proteins such as, e.g., *MYBPC3* encoding myosin binding protein C3 [11]. Due to the amount and size of HCM-associated genes, next generation sequencing (NGS) techniques are frequently used in genetic diagnostics. In this context, the work of Fernlund et al. showed the impact of broad exome sequencing in combination with a virtually defined cardiomyopathy gene panel covering the 60 most likely genes by reanalyzing cases with pediatric cardiomyopathy. The authors revealed pathogenic or likely pathogenic mutations and in 30% additional genetic variants of unknown significance in 50% of the cases [12]. HCM can be also associated with syndromic diseases such as Potocki–Schaffer [13] or Noonan syndrome [14]. Caiazza et al. described an interesting case, where the index patient with Noonan syndrome and HCM carries a likely pathogenic variant in *MYBPC3* and in addition a known pathogenic *PTPN11* (protein tyrosine phosphatase non-receptor type 11) mutation [15]. This report underlines the high relevance of broad genetic testing, which is important for the genetic counseling of the patients and their family members even if syndromic features are present. Peripartum cardiomyopathy (PPCM) is a cardiac disease associated with heart failure and systolic dysfunction during pregnancy or in the first months after delivery. In the majority of cases, PPCM is idiopathic. However, a significant amount of cases with PPCM have a genetic etiology overlapping with other cardiomyopathies. Spracklen et al. presented a review in this Special Issue about the genetic factors involved in PPCM [16]. In most cases, the specific molecular and cellular changes of specific cardiomyopathy-associated mutations are unknown or can only be modelled in cell culture or in animal models because of the lack of explanted human myocardial tissue from mutation carriers. Of note, Sielemann et al. used RNA-sequencing to perform detailed transcriptome analyses revealing differentially expressed genes and regulated pathways in explanted human myocardial tissue samples from cardiomyopathy patients with mutations in *TTN* (titin), *LMNA* (lamin A/C), *RBM20* (RNA binding motif protein 20) and *PKP2* (plakophilin-2) [17]. *TTN*, *LMNA* and *RBM20* are the major DCM genes [18,19,20] and mutations in *PKP2* are common in patients with ACM [21]. Interestingly, the gene expression signatures differ significantly in these four genetic cardiomyopathies indicating a complex and specific remodeling process in the diseased human hearts depending on the specific genotype [17]. However, the clinical cardiac phenotypes associated with mutations in specific genes can be broad. For example, *RBM20* mutations can cause DCM [22], ACM [23] or LVNC [24]. In this context, Vakhrushev et al. report a *RBM20* variant identified in a patient with ventricular arrhythmia but without further structural abnormalities, broadening the clinical spectrum of *RBM20*-associated cardiomyopathies [25]. RBM20 is a cardiac splicing factor and is involved in the splicing of several different cardiac genes such as *TTN* and *RYR2* (ryanodine receptor 2) [26,27]. Another example of a gene causing broad cardiac presentations is *DES*, encoding the muscle specific intermediate filament protein desmin. Desmin filaments connect different multiple-protein complexes and cell organelles such as the desmosomes, Z-bands, mitochondria and nuclei [28]. *DES* mutations were found in patients with skeletal myopathies [29] or with different cardiomyopathies such as DCM [30], ACM [31,32] and RCM [33,34]. Kulikova et al. reported in this Special Issue an LVNC family carrying the *DES* missense mutation p.A337P, leading to a severe filament assembly defect in transfected cells [35]. This genetic finding is in good agreement with further reports of other groups identifying *DES* as a novel LVNC gene [36,37,38]. In contrast to monogenic inherited cardiomyopathies, AF is a complex heart rhythm disorder caused by environmental and multi-genic risk factors [39] and significantly increases the risk for stroke and heart failure. Apixaban is an anticoagulant drug used to reduce the risk for stroke in patients with non-valvular AF [40]. The work of Rosian et al. showed that there was no significant association between peak apixaban plasma concentrations in patients with specific SNPs in the *ABCB1* gene, encoding an ATP-binding cassette (ABC) transporter [41].

In summary, in this Special Issue, different genetic and genomic studies are summarized, describing the impact of genetic factors for different cardiovascular diseases. In addition, three transcriptome analyses of human aortic valves, the ascending aortic tissue and myocardial tissue of mutation carriers with specified genetic cardiomyopathies revealed interesting molecular gene expression patterns in health and disease.
